# Changes in AZGP1 Serum Levels and Correlation With Pulse Wave Velocity After Kidney Transplantation

**DOI:** 10.3389/fcvm.2021.692213

**Published:** 2021-07-05

**Authors:** Thomas Daniel Kraemer, Inga Soerensen-Zender, Nima Memaran, Hermann Haller, Anette Melk, Bernhard Magnus Wilhelm Schmidt, Roland Schmitt

**Affiliations:** ^1^Department of Nephrology and Hypertension, Hannover Medical School, Hanover, Germany; ^2^Department of Pediatric Kidney, Liver, and Metabolic Diseases, Hannover Medical School, Hanover, Germany

**Keywords:** AZGP1, ZAG, arterial stiffness, kidney transplantation, cardiovascular disease, pulse wave velocity

## Abstract

**Background:** Zinc-alpha 2-glycoprotein (AZGP1), a secreted protein with ubiquitous tissue expression, has been controversially linked to the risk of cardiovascular disease. In a cohort of kidney transplant recipients, we measured serum AZGP1 levels after transplantation over a 2 year period and tested for an association with pulse wave velocity as an important parameter indicating future cardiovascular events.

**Methods:** Annual blood sampling and pulse wave velocity measurements were longitudinally performed in 113 kidney transplant recipients. AZGP1 was measured in serum samples using standard ELISA. Association of AZGP1 with pulse wave velocity was longitudinally assessed during follow up of 2 years by mixed longitudinal modeling.

**Results:** AZGP1 serum levels declined significantly after kidney transplantation. This decline was dependent on allograft function as indicated by inverse correlation with eGFR. When corrected for eGFR multivariable analysis revealed an inverse correlation between AZGP1 and pulse wave velocity. This analysis further showed independent associations of older age, higher blood pressure, and higher calcium phosphate product with higher pulse wave velocity.

**Conclusions:** Improved kidney function after transplantation leads to a decline in AZGP1 serum levels. Independent of kidney function and other cardiovascular risk factors lower AZGP1 levels are associated with higher pulse wave velocity in the 2 years after kidney transplantation. These data suggest that AZGP1 might be a potential biomarker for cardiovascular health and a target for improving cardiovascular outcome.

## Introduction

Cardiovascular disease (CVD) is the leading cause of death in patients with end stage renal disease (ESRD) (1). Although, kidney transplantation confers a clear survival advantage for ESRD patients, transplant recipients still have a markedly elevated risk of progressive CVD (2), due to their exposure to classical risk factors (diabetes, hypertension, dyslipidemia) as well as additional risk factors, such as transplant dysfunction, chronic inflammation and exposure to immunosuppressive therapy (3, 4). Given the high CVD burden in kidney transplant recipients it is of great interest to decipher the underlying mechanisms, establish prediction tools and evaluate biomarkers which allow better functional understanding and risk stratification.

Zinc-alpha 2-glycoprotein, AZGP1 (often also abbreviated as ZAG) is a secreted 43 kDa protein which is expressed by many tissues leading to a serum concentration of 30–70 μg/ml in healthy individuals. Despite a multitude of possible implications in different diseases, understanding of this protein's definitive role is still lacking. AZGP1 has been suggested to modify metabolic functions, blood pressure (BP), cancer metastasis, neurological disease and CVD (5–10). As AZGP1 is partially cleared by the kidney, increased serum levels can be observed in patients with kidney dysfunction (11–13). Importantly, there is a discrepancy in reported effects of AZGP1 on CVD between patients with and without chronic kidney disease (CKD). While higher AZGP1 levels were associated with reduced incidence of coronary heart disease and atherosclerosis in non-CKD patients (14, 15), higher AZGP1 levels were shown to correlate positively with CVD in ESRD patients (16). It is therefore interesting, that so far, AZGP1 has not been investigated in the context of kidney transplantation.

Here, we first examined whether serum AZGP1 levels change after kidney transplantation. Secondly, we tested the correlation between AZGP1 levels and pulse wave velocity (PWV) as a measure of arteriosclerosis and cardiovascular health at the time of transplantation and 1 and 2 years afterwards.

## Materials and Methods

This single-center longitudinal-retrospective study included 113 patients from a previously well-characterized cohort of kidney transplant recipients (17). Patients received a kidney allograft at the transplant center of Hannover Medical School between January 2014 and June 2016. At the time of transplantation and during annual follow-up visits (379.1 ± 90.0 and 781.5 ± 116.2 days post-transplant) blood samples were taken and PWV analysis was performed. Data on underlying disease, transplantation history, previous treatments, and current medication were taken from the medical charts and/or by patient interview. The study was approved by the institutional review board (#504) and performed according to the Declaration of Helsinki. All patients gave written informed consent.

### CV Risk Factors Determination and Definition

Height and weight were measured and body mass index (BMI) was calculated. BP measurements were carried out by an automated sphygmomanometer (DINAMAP V100; GE Healthcare) after a resting phase of 5 min. Hypertension was defined as systolic office BP values ≥ 130 mmHg and/or diastolic BP ≥ 80 mmHg and/or current antihypertensive drug treatment. Normotension was defined as BP < 130/80 mmHg without antihypertensive treatment, controlled hypertension as BP < 130/80 mmHg with treatment, uncontrolled hypertension as BP ≥ 130/80 mmHg with treatment, and untreated hypertension as BP ≥ 130/80 mmHg without treatment. Dyslipidemia was defined as total cholesterol ≥ 200 mg/dl and/or low-density lipoprotein (LDL) ≥ 130 mg/dl and/or high-density lipoprotein (HDL) < 40 mg/dl in men and HDL < 50 mg/dl in women (18). Residual renal function was defined by the use of diuretics while on dialysis.

### Laboratory Measurements

Blood and urine samples were analyzed in a central laboratory (Synlab, Heidelberg, Germany) for full blood count, electrolytes, creatinine, cystatin C, total cholesterol, high-density lipoprotein cholesterol (HDL), low-density lipoprotein cholesterol (LDL), high-sensitive C-reactive protein, and parathyroid hormone. The estimated glomerular filtration rate (eGFR) was calculated by using the creatinine-based CKD-EPI formula (19). AZGP1 (μg/ml) was measured in serum samples after storage at −80°C in 113 individuals using a commercial enzyme-linked immunosorbent assay (Biovendor, Modrice, Czech Republic), according to the manufacturer's instructions. Investigators were blinded to patients' data and all measurements were performed in duplicate. The assay sensitivity was 0.673 ng/ml. The intra-assay coefficient of variation was <5%.

### Pulse Wave Velocity

Carotid-femoral PWV was evaluated according to the recommendations of the Task Force III on clinical applications of arterial stiffness using the oscillometric Vicorder System (Skidmore Medical Limited, Bristol, UK; software Ver. 4.0) (20, 21). Measurements were performed in triplicates, with at least 10 heart cycles per measurement. The mean of the three measurements was used for further analysis.

### Statistical Analysis

The statistical analysis was performed using SPSS Version 24.0 (IBM, New York). Data are presented in means ± standard deviation or percentage and absolute numbers. Continuous variables were compared by *t*-test or ANOVA and correlation was evaluated with Spearman correlation coefficient. Categorical variables were analyzed by Chi-squared test. We investigated the association of serum AZGP1 with PWV using longitudinal mixed models during the follow up of 2 years after transplantation. A *P*-value < 0.05 was considered statistically significant.

## Results

Clinical characteristics of the study cohort are presented in Table 1. A total of 113 patients (65 % male, *n* = 73) aged 51.1 ± 14.7 years were enrolled (age range 18–78 years). At the time of transplantation mean BMI was 26.6 ± 4.3 kg/m^2^ (range 17.4–37.2). Forty-nine percent (*n* = 55) had been diagnosed with CVD (coronary artery disease, post myocardial infarction, chronic heart failure, left ventricular hypertrophy, atrial fibrillation, peripheral artery disease, or a history of stroke). Twenty percent (*n* = 23) suffered from diabetes mellitus and 96 % (*n* = 108) had arterial hypertension. The dialysis vintage mean was 80.6 ± 45.9 months (range 12–198). Of all recipients, 85 % (*n* = 96) had not undergone a previous transplantation and 19 % (*n* = 21) were preemptively transplanted.

**Table 1:** Clinical characteristics of the study cohort (*n* = 113) at the time of transplantation.

**Clinical characteristics**
**Sex**	
Female	40/113 (35%)
Male	73/113 (65%)
Age (years)	51.1 ± 14.7 (18–78)
Body mass index (kg/m^2^)	26.6 ± 4.3 (17.4–37.2)
Cardiovascular diagnosis	55/113 (49%)
Hypertension	108/113 (96%)
Dyslipidemia	11/113 (10%)
Diabetes mellitus	23/113 (20%)
**Underlying renal disease**	
Diabetes	14/113 (12%)
Vascular	18/113 (16%)
Glomerulonephritis	38/113 (34%)
Interstitial	5/113 (4%)
Hereditary	24/113 (21%)
Systemic	4/113 (4%)
Unknown	5/113 (4%)
Others	14/113 (12%)
Dialysis vintage (months)	80.6 ± 45.9 (12–198)
Peritoneal dialysis	7/113 (6%)
Hemodialysis	85/113 (75%)
Preemptive Tx	21/113 (19%)
Living donation	37/113 (33%)
**Immunosuppression**	
Calcineurin inhibitors	110/113 (97%)
mTOR inhibitors	14/113 (12%)
Mycophenolic acid	110/113 (97%)
Steroids	112/113 (99%)

*Continuous values are given as mean ± standard deviation with the range in brackets. Categorical values are given as numbers and percentages. The denominator shows the number of available observations for each parameter, and the numerator indicates the number of patients who fulfill that measure. Cardiovascular diagnosis include coronary artery disease, post myocardial infarction, chronic heart failure, left ventricular hypertrophy, atrial fibrillation, peripheral artery disease, or a history of stroke. mTOR, mammalian target of rapamycin*.

**Figure 1:**
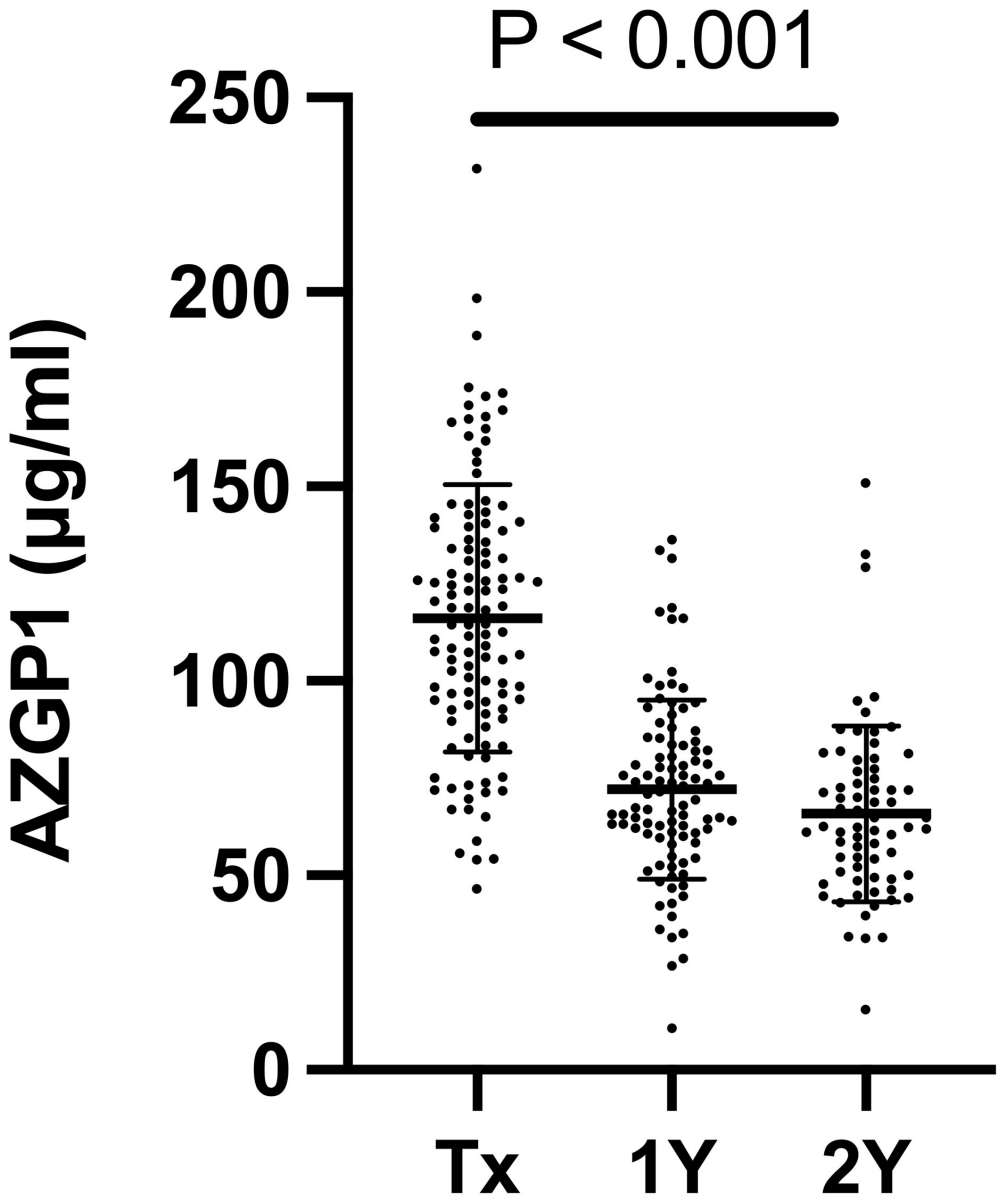
Serum AZGP1 levels of kidney transplant recipients at the time of transplantation (Tx), 1 year (1Y) and 2 years (2Y) after transplantation. *n*_Tx_ = 113, *n*_1Y_ = 97, *n*_2Y_ = 69. Graph shows individual values with means and corresponding standard deviations. Groups were compared by mixed-effects analysis.

Mean AZGP1 serum levels were 116 ± 34.4 μg/ml (range 46.6–231.8, *n* = 113) at the time of transplantation. We found no correlation between AZGP1 serum levels and underlying renal disease (*P* = 0.654), preemptive vs. non-preemptive transplantation (*P* = 0.804), dialysis modality comparing peritoneal vs. hemodialysis (*P* = 0.967) and residual renal function while on dialysis (*P* = 0.264). During the follow up of 2 years serum AZGP1 levels dropped significantly (Figure 1). One year after transplantation, AZGP1 levels decreased by 38 % (72 ± 23.1 μg/ml, range 10.7–136.4, *n* = 97) and by 43 % (66 ± 22.6 μg/ml, range 15.6–150.9, *n* = 69) at 2 years post-transplant (*P* < 0.001, Figure 1). Correlation analysis confirmed an inverse correlation of AZGP1 with renal allograft function as estimated by eGFR (CKD-EPI, creatinine) at the time of transplantation (*r* = −0.235, *P* = 0.012), after 1 year (*r* = −0.470, *P* < 0.001) and after 2 years (*r* = −0.246, *P* = 0.045, Table 2). For all other investigated parameters, including PWV, correlation effects were negligible, or interpreted as clinically irrelevant.

**Table 2:** Spearman correlations of AZGP1 serum levels and indicated variables for the corresponding time points.

**Variable**	**At the time of transplantation**			**1 Year follow-up**			**2 Years follow-up**		
	**r**	***P*-value**	***n***	**r**	***P*-value**	***n***	**r**	***P*-value**	***n***
Recipient age (years)	0.058	0.545	113	0.143	0.163	97	0.1	0.411	69
Dialysis vintage (months)	0.157	0.136	92	0.019	0.871	77	0.145	0.302	53
Body mass index (kg/m^2^)	−0.026	0.785	113	−0.151	0.167	85	−0.069	0.577	67
eGFR (ml/min/1.73 m^2^)	−0.235^*^	**0.012**	113	−0.470^**^	** <0.001**	96	−0.246^*^	**0.045**	67
Calcium (mmol/L)	−0.095	0.32	111	−0.095	0.362	94	−0.161	0.197	66
Phosphorus (mmol/L)	0.112	0.241	111	0.127	0.223	94	−0.124	0.323	66
PTH (pg/ml)	0.125	0.192	111	0.226^*^	**0.029**	94	0.19	0.133	64
Albumin (g/L)	0.114	0.233	111	−0.004	0.97	94	−0.088	0.481	66
CRPHS (mg/L)	−0.174	0.067	111	0.076	0.465	94	0.192	0.123	66
Cholesterol (mg/dl)	0.077	0.425	111	0.240^*^	**0.02**	94	0.226	0.068	66
HDL (mg/dl)	−0.012	0.897	111	0.072	0.489	94	−0.011	0.929	66
LDL (mg/dl)	0.156	0.102	111	0.094	0.367	94	0.194	0.119	66
PWV (m/s)	−0.157	0.105	107	−0.098	0.382	82	−0.077	0.554	62

*eGFR, estimated glomerular filtration rate; PTH, Parathyroid hormone; CRPHS, high sensitive C-reactive protein; HDL, High density lipoprotein cholesterol; LDL, Low density lipoprotein cholesterol; PWV, Pulse wave velocity*.

* *P < 0.05,*

** *P < 0.01.*

*Variables with P < 0.05 are in bold*.

We performed a longitudinal mixed model analysis of PWV and AZGP1 alone (Model A), AZGP1 together with eGFR (Model B) and AZGP1 together with eGFR and other known modifiers of arterial stiffness (age, sex, BP, and calcium-phosphorus product; Model C) (Table 3). While there was no significant association in Model A, AZGP1 and eGFR, both, had a significant negative association with PWV in Model B. This effect remained stable for AZGP1 in Model C, indicating an inverse correlation between AZGP1 and PWV. The final multivariate model (Model C) revealed lower AZGP1, older age, higher systolic BP and higher calcium phosphorus product to be independently associated with higher PWV. eGFR showed a trend for negative association with PWV in Model C (*P* = 0.08).

**Table 3:** Longitudinal mixed model analysis of PWV and AZGP1 alone (Model A), AZGP1 together with eGFR (Model B) and AZGP1 together with eGFR, age, sex, blood pressure, and calcium-phosphorus product during the follow up of 2 years after transplantation.

**Mixed Model analysis of PWV**									
**Variable**	**Model A**			**Model B**			**Model C**		
	**Estimate**	**SE**	***P*****-value**	**Estimate**	**SE**	***P*****-value**	**Estimate**	**SE**	***P*****-value**
Intercept	7.3891	0.2638	<0.0001	8.1501	0.3528	<0.0001	1.9669	0.8467	0.0210
Time since Tx (days)	0.000594	0.000447	0.1851	0.000769	0.000443	0.0838	**0.000721**	**0.000337**	0.0334^*^
AZGP1 (μg/ml)	−0.00316	0.002357	0.1807	**−0.00811**	**0.002790**	0.0040^**^	**−0.01031**	**0.002792**	0.0003^***^
eGFR (ml/min/1.73 m^2^)				**−0.01879**	**0.005908**	0.0016^**^	−0.00874	0.004827	0.0805
Age (years)							**0.05682**	**0.005190**	<0.0001^***^
Sex (male vs. female)							0.1016	0.1627	0.5329
Systolic BP (mmHg)							**0.02116**	**0.004499**	<0.0001^***^
Ca*PO4 product							**0.2169**	**0.09065**	0.0175^*^

BP, Blood pressure; Ca*PO4, Calcium phosphorus product; eGFR, estimated glomerular filtration rate; PWV, Pulse wave velocity; Tx, Transplantation; SE, Standard error.

* *P < 0.05,*

** *P < 0.01,*

*** *P < 0.001.*

*Variables with P < 0.05 are in bold*.

## Discussion

Although kidney transplantation offers clear survival benefits compared to dialysis, CVD remains one of the leading causes of premature death after transplantation (22). As AZGP1 has been identified as a potential modifier of CVD (14–16), we first assessed serum AZGP1 changes after kidney transplantation over a period of 2 years. We found a continuous decline in AZGP1, which paralleled eGFR improvement. This finding is consistent with the concept that better kidney function reduces the protein's half-life (23). Additionally, the uremic milieu may stimulate overproduction of AZGP1 in white adipose tissue (24). However, the contribution of adipose tissue to systemic AZGP1 is questionable, because adipocyte secretion is mainly local (25). There was no clear correlation between AZGP1 and BMI, total cholesterol, LDL, or HDL, which is consistent with previous data by us and others suggesting that the postulated adipokine role of AZGP1 might be less relevant in CKD patients (11, 13, 26).

Secondly, we determined the potential relevance of AZGP1 as a biomarker for CVD prediction after transplantation. For this purpose we investigated whether AZGP1 levels correlated with PWV development as a surrogate parameter for arterial stiffness over 2 years after transplantation. Our analysis revealed a significant inverse association of AZGP1 with PWV. Importantly, this association was only observed when corrected for eGFR. While this pattern is compatible with previous observations in non-CKD patients showing a correlation between higher AZGP1 levels and reduced incidence of coronary heart disease and atherosclerosis scores (14, 15), it is in conflict with findings in dialysis patients where higher AZGP1 levels positively correlated with cardiovascular events and mortality (16). This discrepancy could be explained by the dominating effects of non-traditional uremia associated risk factors in ESRD patients as opposed to a more traditional risk factor constellation in patients after kidney transplantation (27). According to this notion, our data suggest that the beneficial role of AZGP1 might be restored in transplant recipients. Strategies to increase AZGP1, such as pharmacologic inhibition of sodium-glucose cotransporter 2 (28), could therefore be an option to improve cardiovascular health and clinical outcome of kidney transplant recipients and should be tested in future studies.

We recognize several limitations inherent to our study: Due to the observational study design we cannot draw conclusions about potential cause-effect relationships. Moreover, findings might not be transferable to other transplant centers and more diverse populations because this is a single-center study with a limited patient number and follow-up period. Another limitation of our study is the lack of clinical endpoints. Although PWV has been validated in transplant patients (29, 30) as a surrogate endpoint for CVD, longitudinal PWV data are conflicting, describing a decrease (31), no change (29), or –as in our cohort- an increase (32, 33) after transplantation. It has been suggested that this heterogeneity might be due to differences in follow-up time, showing a decrease in shorter studies and an increase in longer studies (34). Taking these considerations into account, our findings need to be validated in a multi-center study with clinical CV outcome events over a longer period of time to draw stronger conclusions and confirm if AZGP1 can be a useful biomarker.

In summary, our study provides valuable novel results: (1) Kidney transplantation reduces AZGP1 concentrations which are elevated in ESRD. (2) Lower AZGP1 is independently associated with higher PWV in renal transplant recipients. (3) Protective effects of AZGP1 on the cardiovascular system seem to be restored after kidney transplantation. Together, this suggests that AZGP1 might be a potential biomarker for cardiovascular health in kidney transplant recipients and that it should be explored as a target for improving cardiovascular outcome.

## Data Availability Statement

The raw data supporting the conclusions of this article will be made available by the authors, without undue reservation.

## Ethics Statement

The studies involving human participants were reviewed and approved by Hannover Medical School Institutional Review Board (#504). The patients/participants provided their written informed consent to participate in this study.

## Author Contributions

TK, AM, BS, and RS conceived the study and wrote the manuscript. IS-Z conducted AZGP1 measurements. NM conducted PWV measurements. TK and BS performed bioinformatics analysis. TK, BS, and RS interpreted results with contributions of AM and HH. All authors approved the final version of the manuscript.

## Conflict of Interest

BS has received lecture fees from Berlin Chemie-Menarini, Daichii-Sankyo, BMS, Pfizer, MSD. RS has received lecture and consulting fees from FMC and Otsuka. HH has received lecture fees, advisory board Consultant fees, and Research funds from Boehringer Ingelheim, Bayer and Astra Zeneca. The remaining authors declare that the research was conducted in the absence of any commercial or financial relationships that could be construed as a potential conflict of interest.
